# FlyPhy: a phylogenomic analysis platform for *Drosophila *genes and gene families

**DOI:** 10.1186/1471-2105-10-123

**Published:** 2009-04-25

**Authors:** Jinyu Wu, Xiang Xu, Jian Xiao, Long Xu, Huiguang Yi, Shengjie Gao, Jing Liu, Qiyu Bao, Fangqing Zhao, Xiaokun Li

**Affiliations:** 1 Institute of Biomedical Informatics/Zhejiang Provincial Key Laboratory of Medical Genetics, Wenzhou Medical College, Wenzhou 325000, PR China; 2 School of Pharmaceutical Science/Zhejiang Provincial Key Laboratory of Biotechnology Pharmaceutical Engineering, Wenzhou Medical College, Wenzhou 325035, PR China; 3 Department of Biochemistry and Molecular Biology, Pennsylvania State University, Pennsylvania 16802, USA

## Abstract

**Background:**

The availability of 12 fully sequenced *Drosophila *species genomes provides an excellent opportunity to explore the evolutionary mechanism, structure and function of gene families in *Drosophila*. Currently, several important resources, such as FlyBase, FlyMine and DroSpeGe, have been devoted to integrating genetic, genomic, and functional data of *Drosophila *into a well-organized form. However, all of these resources are gene-centric and lack the information of the gene families in *Drosophila*.

**Description:**

FlyPhy is a comprehensive phylogenomic analysis platform devoted to analyzing the genes and gene families in *Drosophila*. Genes were classified into families using a graph-based Markov Clustering algorithm and extensively annotated by a number of bioinformatic tools, such as basic sequence features, functional category, gene ontology terms, domain organization and sequence homolog to other databases. FlyPhy provides a simple and user-friendly web interface to allow users to browse and retrieve the information at multiple levels. An outstanding feature of the FlyPhy is that all the retrieved results can be added to a workset for further data manipulation. For the data stored in the workset, multiple sequence alignment, phylogenetic tree construction and visualization can be easily performed to investigate the sequence variation of each given family and to explore its evolutionary mechanism.

**Conclusion:**

With the above functionalities, FlyPhy will be a useful resource and convenient platform for the *Drosophila *research community. The FlyPhy is available at .

## Background

Fruit flies have been studied for many years and one species of them, in particular *D. melanogaster*, is a very important model organism for understanding genetic, developmental, cellular, ecological, and evolutionary processes. The sequencing of *D. melanogaster *and *D. pseudoobscura *genome, first resealed in 2000 and 2005, respectively, provide significant contributions to the fruit fly biology and genome research [1,2]. With ever-developing large-scale sequencing technologies, 12 *Drosophila *genomes are available and accessible online now [3,4]. The availability of these *Drosophila *genomes offers an unprecedented opportunity to explore the evolution of *Drosophila *gene families, which can serve as a significant base for functional genomics and provide an important advance for understanding sequence-structure-function relationships of *Drosophila *genes among different species. For example, comparative genomics analysis revealed that there was a high-frequency occurrence of gene gain and loss in *Drosophila *gene families, even among closely related *Drosophila *species [5]. Genome-wide comparison of immune-system genes in *Drosophila *revealed that, in contrast to signaling proteins, effector proteins are much more likely to vary in copy number across different *Drosophila *species [6]. Based on phylogenomic approaches, the possible reason for the origin of new genes and subsequent lineage-specific evolution at different time nodes in the *Drosophila *is well revealed from a genome-wide level [7].

Development of effective and integrated bioinformatics databases and tools is an important work for facilitating more rapid progress in *Drosophila *research, which will provide a convenient aid to *Drosophila *research communities. In support of this, several databases have been devoted to *Drosophila *in a well-organized form. FlyBase is a premier public database with integrated genetic, genomic, and functional data of *Drosophila*[8]. FlyMine is a comprehensive database with gene expression data of *Drosophila *[9] and DroSpeGe is a genome database with comparative annotations of 12 *Drosophila *species [3]. Other resources such as Berkeley Drosophila Genome Project [10] and AAA [11] are also useful resources for *Drosophila *biologists. However, all of these available resources are gene-centric. The Dfam database contains descriptions of the families, alignments, gene trees [5], but there is no integrated database to provide comprehensive information on gene families of *Drosophila*. Comparative genomics and molecular evolution analysis of *Drosophila *gene families has been demonstrated to be a powerful approach to study their evolution, structure and function. In this study, we applied a graph-based Markov Clustering algorithm to classify all the *Drosophila *proteins into families. Thereby, a comprehensive platform containing putative protein families with extensive annotation information of 12 fruit fly species was developed. Users can easily interact with the protein families of their interest and other relevant detail annotations of genes by browsing, keyword searching or BLASTing. Through the workset, the retrieved data can be well integrated for phylogenomic analysis.

## Construction and content

### Protein family clustering and detailed annotation

**Figure 1:**
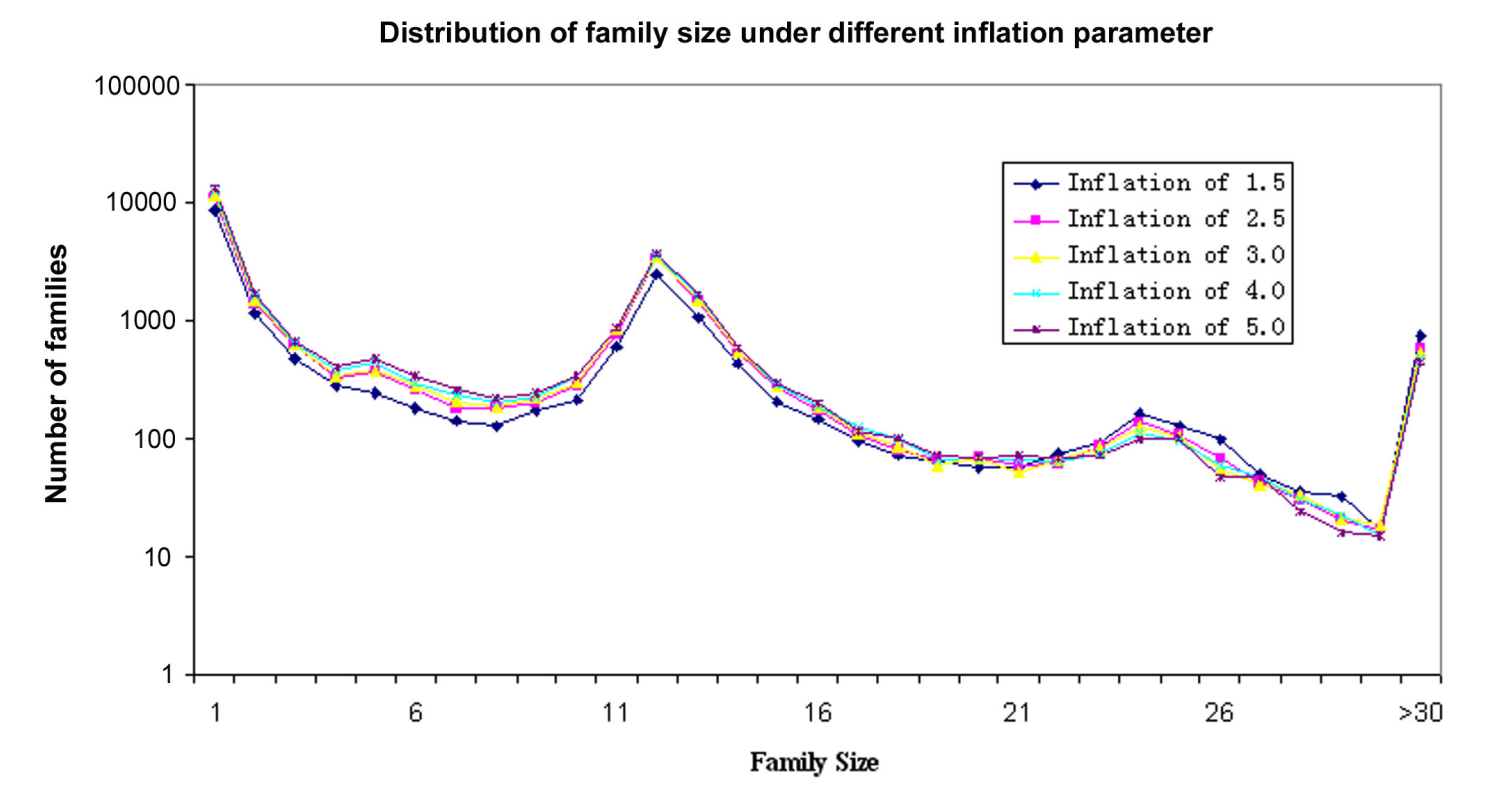
**The distribution of family size under different inflation parameter of 1.5, 2.5, 3.0, 4.0 and 5.0 in the TribeMCL program**.

Protein families can be expressed as a group of proteins that share significant similarity in sequence and have a common evolutionary history. In comparison with other tools for grouping different proteins into putative families, such as BLASTClust (from the NCBI BLAST suite) and cd-hit program [12], the TribeMCL [13] has been proved to be a good alternative choice for clustering of divergent proteins and thus has wide applications [14-16]. The TribeMCL program first calculates the pair-wise distances between all genes from the genomes and then uses a graph-based Markov Clustering algorithm to generate clusters. With such algorithm, it can effectively break the barriers during the clustering process, such as multi-domains, fragments of proteins and promiscuous domains in the alignment. The main parameter that influences the number and size of clusters in the TribeMCL program is the inflation value, which defines the tightness of the clustering results [13]. In this study, we used the TribeMCL program to generate protein families from 181, 780 protein sequences of 12 *Drosophila *species [17]. An all-against-all BLASTP was performed for the protein sequences using the BLAST program with an E-value of 1e-5. Finally, putative protein families were generated using the TribeMCL program with multiple inflation values (1.5, 2.5, 3.0, 4.0 and 5.0 respectively). We found that different inflation parameters produced similar results and the cluster size varied greatly in different families (Figure 1), indicating the frequent gene gain-and-loss in *Drosophila*. As expected, a large number of clusters with a family size of 12 were observed and the majority of them represented families with only one gene in each species. For example, among the 3,454 families (family size = 12) obtained from the inflation value 3, a total of 3,055 families had a one-to-one orthologous relationship. It is suggested that these families may represent a core set of genes and universally present in different *Drosophila *species.

Then these genes and gene families were extensively annotated based on a number of bioinformatic tools and databases. In particular, the PepStat program implemented in the EMBOSS package was used to predict the molecular weight and isoelectric point of a given protein [18]. The InterProScan program was used to assign gene domain architectures against integrated databases, including PROSITE, PRINTS, Pfam, ProDom, SMART, TIGRFAMs, PIRSF and SUPERFAMILY. The InterProScan results were mapped to Gene Ontology terms, including cellular component, biological process and molecular function, using the InterPro2Go [19]. BLAST searches were performed against several major databases, such as PDB (collected on 18 December 2008), Uniprot (release 14.6) and Refseq (release 32). The best hit from the Uniprot database was used as a controlled vocabulary for the description of gene function. In addition, the functional categorization of all genes and protein families was carried out by BLASTing to the COG and KEGG databases with an E-value of 1e-5. In order to annotate each cluster, we obtained the annotation information in KEGG pathway related to each member in the clusters, and then manually curated the most common description of the members in each cluster and assign them to the clusters.

### Database construction

The design scheme of FlyPhy is similar to our previous integrated pipeline of ArchaeaTF [20] and, PlasmoGF [15], which is constructed based on open source software, including Apache, MySQL, PHP and Perl, etc. The curated data of gene families, as well as various annotation information, are stored in a MySQL database system and can be accessed using Structure Query Language (SQL). The web platform is base on Apache HTTP server and its pages are generated via a combination of PHP language and Perl CGI scripts. Meanwhile, the BioPerl modules are applied to manipulate data and convert different data formats. All the procedures above are executed on the Linux operating system.

## Utility and discussion

### Data retrieving

**Figure 2:**
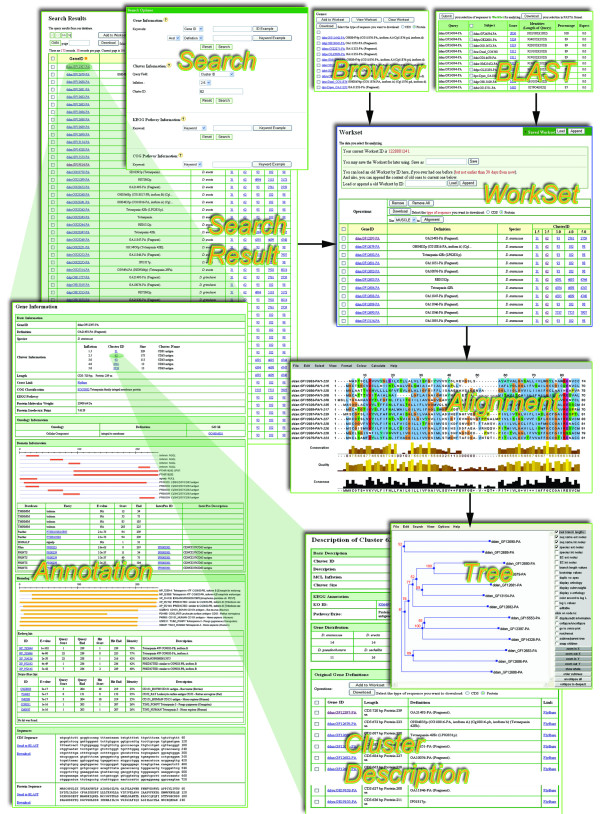
**The snapshot indicating the interrelationship of FlyPhy data and tools**. The annotated information can be retrieved at multiple levels: keyword search, browse and BLASTing. The retrieved results will be shown in a table with the basic information of each gene or gene family matching the query and further linked to the detailed annotation of the gene and gene family also is also provided. Meanwhile, users can construct their own workset to investigate the sequence variation of each given family and explore its evolutionary mechanism.

FlyPhy provides a simple and user-friendly interface for researchers to access gene and gene family data (Figure 2). Users can browse and search (both keyword search and sequence similarity search) all the data at different levels by the integrated functions.

#### Browse

all the genes have been organized into different functional categories according to the COG and KEGG database. Individual COG category can be browsed easily in the COG browser as well as the list of genes classified under each specific COG category. The KEGG pathway can be explored in the same way in the KEGG browser. Clicking on the gene ID will show its detailed annotation information, such as basic sequence features, Gene Ontology terms, gene domain organization and sequence homolog to other relevant databases.

#### Keyword search

FlyPhy provides a powerful multi-layered query system. Firstly, in the search page, users can search genes by keywords (gene ID or gene definition), or by clusters under different inflation parameters (eg: cluster ID, cluster size and cluster definition). Meanwhile, the functional categories of different gene family can also be retrieved by KEGG or COG ID and keyword. The search results will be shown in table with the basic information of each gene matching the query. In the table, the IDs of each gene and its clusters are linked to the detailed annotation of the gene and clusters.

#### Sequence similarity search

We have generated genes and protein databases for all the families, and enabled protein or nucleotide BLAST in FlyPhy. This facility will help users query and verify the members of a specific gene family based on their own sequences. Further, with the implement of the ViroBLAST program [21], the sequence similarity search of FlyPhy provides many advanced options to allow users to easily parse and manipulate the search results.

### Workset-centric data manipulation and phylogenomic analysis

An important functionality of FlyPhy is that it adopts the workset to organize the genes and protein families (Figure 2). All the retrieved results can be added to the workset for further data manipulation and phylogenomic analysis. Each workset is assigned with a specific ID either generated by the server randomly or saved as a user's own favorite name. All the data in the workset can be customized through appending or deleting the items. More importantly, FlyPhy allows users to load an old workset by ID if they ever had established before to avoid generating the same workset from scratch.

To explore the sequence conservation of the data stored in the workset, multiple sequence alignment can be performed using either the ClustalW [22] or MUSCLE program [23] with user-definable parameters at amino acid or DNA level. Visualization of the aligned results is conducted by the Jalview program [24], which is based on Java Applet (prior to use this program, a Java Runtime Environment is needed on the local computer). To investigate the evolutionary relationship of genes or proteins stored in the workset, users can directly use the QuickTree program to construct a phylogenetic tree [25], which is based on the neighbor-joining algorithm. The reliability of the tree can be evaluated with different replicates of bootstrapping test. The graphical representation of the inferred tree is carried out using the ATV program [26].

## Conclusion

The purpose of constructing FlyPhy is to develop a comprehensive platform on which users can access detailed annotation information, investigate the sequence variation and explore the evolutionary mechanism of each family in *Drosophila*. Through the form of workset, the retrieved data are well integrated, and phylogenomic analysis can be easily performed. In the future, we will continue to collect relevant fly data once fully sequenced genome of other *Drosophila *species is available. More phylogenomic tools will be incorporated to help users reconstruct the evolutionary history of gene families, such as the TREE-PUZZLE program for phylogenetic tree construction based on maximum likelihood algorithm and the PAML program for evolutionary rate inference. We believe that FlyPhy will serve as a useful platform for *Drosophila *biologists and relevant researchers to study the comparative genomics and phylogenomics of fly gene families efficiently and conveniently.

## Availability and requirements

***Project name***: FlyPhy: a Phylogenomic Analysis Platform for *Drosophila *Genes and Gene Families

***Project home page***: 

*Operating system(s)*

For user: Standard WWW browser, such as Firefox3.0, Internet Explorer7.0 and Safari3.1

For server: Linux

***Programming language***: PHP, MySQL, Perl and BioPerl

***License***: GNU GPL

***Any restrictions to use by non-academics***: None

## Authors' contributions

JW and XX performed bioinformatics analysis, constructed the database, developed the web interface, and wrote the draft manuscript. JX provided scientific suggestions and criticisms for improving the manuscript and website. LX, HY, SG and JL participated in the data analysis and the update of the database. XL, FZ and QB participated in its design, helped write the manuscript and supervised the whole project. All authors read and approved the final manuscript.
